# Successful emotion regulation *via* cognitive reappraisal in authentic pride: Behavioral and event-related potential evidence

**DOI:** 10.3389/fnhum.2022.983674

**Published:** 2022-10-13

**Authors:** Daichun Lin, Jianru Bi, Xiaoxuan Zhang, Feng Zhu, Yanmei Wang

**Affiliations:** ^1^Shanghai Key Laboratory of Mental Health and Psychological Crisis Intervention, School of Psychology and Cognitive Science, East China Normal University, Shanghai, China; ^2^Shanghai Changning Mental Health Center, Shanghai, China

**Keywords:** authentic pride, hubristic pride, cognitive reappraisal strategy, down-regulation of negative emotions, event-related potentials

## Abstract

The present study explored whether authentic pride (AP) and hubristic pride (HP) were differently associated with cognitive reappraisal strategy. In study 1, undergraduates (*n* = 235) completed a battery of self-report questionnaires, including the Authentic and Hubristic Pride-Proneness Scale (AHPPS), Emotion Regulation Questionnaire (ERQ), and emotion regulation questionnaire (ERP-R). The results showed that AP significantly predicted successful down-regulation of negative emotions *via* a spontaneous cognitive reappraisal strategy. However, hubristic pride (HP) was negatively associated with spontaneous cognitive reappraisal. In study 2, participants with trait AP (*n* = 31) and trait HP (*n* = 29) undergoing continuous electroencephalogram (EEG) recording were required to reinterpret emotional pictures to down-regulate/up-regulate their negative/positive emotional reactions. The results showed that individuals with AP reported lower levels of emotional arousal and lower amplitudes of late positive potentials (LPPs) than did individuals with HP in response to negative pictures during the down-regulation of negative emotions, but not during passive viewing or up-regulation of positive emotions. Across two studies, these findings showed that individuals with AP could utilize the cognitive reappraisal strategy (spontaneously in daily life and under experimental instructions) to down-regulate negative emotions more successfully relative to individuals with HP.

## Introduction

Pride is a type of positive emotion, and the feeling of pride is related to one’s own accomplishments (Tracy and Robins, 2007a,^2014^). As a self-conscious emotion related to humans’ social status and group acceptance (Shariff and Tracy, 2009), pride plays a critical role in many domains of human behavior or psychological functioning (e.g., Oveis et al., 2010; Shimoni et al., 2016). Researchers have distinguished pride into two different facets: authentic pride (AP) and hubristic pride (HP) (Tracy and Robins, 2007b,^2014^). According to past research, AP is related to genuine feelings of self-worth, and occurs when one attributes success to internal, unstable, and controllable reasons, while HP is associated with self-aggrandizing motives and occurs when an individual attributes success to internal, stable, and uncontrollable reasons (Tracy and Robins, 2007b; Tracy et al., 2009). Thus, there might be two kinds of individuals: one more likely to experience AP and the other more likely to experience HP. Researchers have found that different types of pride have been linked to various highly divergent outcomes. AP is more likely to be associated with positive behaviors such as self-control, perseverance, agreeableness, conscientiousness, achievement-oriented behaviors, more effective leadership behaviors, helping behavior, and moral behavior, and learning new information and it might contribute to an individual’s well-being (Tracy and Robins, 2007b; Tracy et al., 2009; Carver et al., 2010; Krettenauer and Casey, 2015; Brosi et al., 2016; Van Doren et al., 2019; Yeung and Shen, 2019; Mercadante et al., 2021). However, HP is more likely to be related to maladaptive outcomes, such as aggressive, abusive, and antisocial behaviors, interpersonal conflict, poorer mental health states, and antisocial and dishonest means to achieving high status (McGregor et al., 2005; Carver et al., 2010; Cheng et al., 2010; Orth et al., 2010; Wubben et al., 2012; Yeung and Shen, 2019; Mercadante et al., 2021). Although important strides in understanding the distinct effects of these two facets of pride have been made, comparatively little work has been done to examine the unexplored question of whether authentic and hubristic pride would influence the effectiveness of emotion regulation differently.

Emotion regulation refers to the attempts to influence one’s own emotions (McRae and Gross, 2020) and has been considered essential for an individual’s mental health (Berking and Wupperman, 2012; Desrosiers et al., 2013; Henry et al., 2016), successful social interactions, and well-being (Haga et al., 2009; McRae et al., 2012). Cognitive reappraisal and expressive suppression are two strategies representative of emotion regulation (Gross, 2002; Goldin et al., 2008; Kalokerinos et al., 2015; Pan et al., 2019). Cognitive reappraisal is an example of an antecedent-focused strategy that aims to reinterpret the meaning or self-relevance of a situation or emotional event to alter an emotional response (Gross and John, 2003; Morris et al., 2014). Expressive suppression refers to inhibiting emotional-related behaviors (e.g., making facial expressions) associated with an emotional response and is a type of response-focused strategy (Gross, 1998). Though both strategies can reduce negative emotional responses, cognitive reappraisal has been found to be positively related to subjective well-being, mental health, and lower self-reported stress-related symptoms (e.g., Moore et al., 2008; Sai et al., 2016; Brockman et al., 2017). In contrast, expressive suppression is more frequently used in individuals with high trait anxiety, posttraumatic stress disorder, or attachment anxiety (Sippel et al., 2016; Pan et al., 2019; Girme et al., 2021).

Two distinct facets of pride (AP vs. HP) are characterized by distinct ways of attributing cause to someone’s success (Tracy and Robins, 2007b; Tracy and Prehn, 2012). “Authentic Pride” has been characterized as attributing success to one’s temporary effort, whereas “Hubristic Pride” is characterized as attributing success to one’s stable, uncontrollable, innate ability. Therefore, that individuals with two facets of pride might show distinct behavioral reactions to failure or distinct interpretable styles of negative emotional responses (Brooks et al., 2019; Mercadante et al., 2021). Individuals with trait AP were more likely to reinterpret the reason for failure as a more unstable, controllable cause (temporary effort) and to adopt a positive attitude in the face of failure. For example, individuals with AP might interpret that every failure is an opportunity to learn how to better reach one’s goals and that negative emotional responses can be changed with efforts. In contrast, individuals with HP are more likely to view the failure as a threat to their self-appraisal of having high ability, resulting in maladaptive emotional regulation (Carver et al., 2010). Furthermore, individuals with HP are more likely to experience fear of negative evaluation from others after failure; they may consider negative emotion responses as uncontrollable and difficult to change. A recent study showed that individuals with HP might use the antisocial behavior of dishonesty to gain status when faced with a status threat (Mercadante and Tracy, 2022). Researchers assumed that hubristically proud individuals exaggerated their performance in their competence to gain higher social status than they actually deserve, and they might carry out some tactical behaviors to avoid to feel inferior to others (Stanger et al., 2021; Mercadante and Tracy, 2022). For these reasons, we speculate that individuals with trait HP tend to expressive their positive emotions exaggeratedly and to suppress their negative emotional reactions or negative emotional behaviors to avoid being judged negatively or being despised by others, and then to attain higher social status.

Authentic pride is associated with high intrinsic motivation, self-control, creativity, and the ability to delay gratification, all of which facilitate learning new information (Damian and Robins, 2013; Ho et al., 2016). In contrast, HP is associated with a pattern of traits that are opposite to those associated with AP (Mercadante et al., 2021). Relative to expressive suppression, cognitive reappraisal is more reliant on creativity and the ability to learn new information, both of which are necessary to flexibly reinterpret negative events and down-regulate negative emotions when successfully using this strategy. Therefore, we speculated that individuals with trait AP were more likely to use the cognitive reappraisal strategy to regulate their negative emotions effectively.

### The present study

Taken together, the present research aimed to investigate whether these two forms of pride are differently associated with emotion regulation strategies across two studies. In study 1, we examined whether these two forms of pride are differently related to the self-reported use of emotion regulation strategies (cognitive reappraisal vs. expressive suppression) in everyday life. We hypothesized that individuals with AP would endorse greater habitual use of cognitive reappraisal to down-regulate negative emotional reactions in daily life compared to those with HP; however, HP was expected to be related to greater habitual use of expressive suppression.

Considering the temporal features of emotion regulation, the excellent temporal resolution associated with the event-related potentials (ERPs) technique makes it advantageous for exploring the dynamic time course of emotion regulation. The late positive potential (LPP) is a positive-going deflection in the ERP waveform that begins approximately 300 ms after stimulus onset and lasting for as long as 5 s during the stimulus presentation with maximal magnitude typically at the central-parietal region (Zhang et al., 2019). Previous studies have shown that the late positive potential (LPP) response to emotional stimuli is larger than that to neutral ones (Hajcak and Olvet, 2008; Hajcak et al., 2010; Hartigan and Richards, 2017), and the LPP appears to be sensitive primarily to arousal level rather than valence (Hajcak and Foti, 2020). Because of its sustained duration and its sensitivity to the affective properties of pictorial stimuli, the LPP is particularly well-suited to be a good candidate for studies exploring the electrophysiological correlates of emotion regulation by means of cognitive reappraisal manipulation and has featured most prominently in this work (Krompinger et al., 2008; Cao et al., 2020; Xiao et al., 2021; MacNamara et al., 2022). Specifically, there is evidence showed that cognitive reappraisal strategy could effectively reduce the LPP and subjective ratings of unpleasantness and arousal elicited by negative pictures (Moser et al., 2006; Foti and Hajcak, 2008; Dennis and Hajcak, 2009). Furthermore, the amplitude of the LPP was lower when participants reappraised negative pictures than when they passively viewed negative pictures (Moser et al., 2010; Parvaz et al., 2012). The decrease in LPP amplitude during cognitive reappraisal was associated with a decrease in self-reported negative emotion (Hajcak and Nieuwenhuis, 2006). The reappraisal-related modulation of the LPP has also been replicated in older adults (Meynadasy et al., 2022), and in 5–9 year old children (Myruski and Dennis-Tiwary, 2021). The LPP has also been used to investigate the lasting effects of reappraisal (Bautista et al., 2022). Consequently, the change in LPP amplitude can be considered as an important neurological indicator of online reappraisal facility (Kinney et al., 2019).

To our knowledge, very few studies have explored the temporal neural processing of AP and HP during experimental emotion regulation tasks. Therefore, we used ERPs in study 2 to investigate the neural correlates of emotion regulation in individuals with authentic and hubristic pride. Participants were instructed to passively view emotional pictures, reappraise negative pictures to reduce negative emotion (down-regulation), or reinterpret positive pictures to increase positive emotion (up-regulation). ERPs and self-reported measures of emotional reactions were recorded. We focused on LPP as the neurologic index of successful emotion regulation. We hypothesized that individuals with AP reported lower emotional intensity to negative pictures during reappraisal relative to those with HP.

Although using self-report measures is a traditional method to study emotion regulation or emotional response, neural markers may provide more direct access to emotional arousal, and its regulation, while avoiding many of the potential pitfalls. Thus, it may be that both the ERP and self-report data are providing accurate information about emotion regulation (Hampton et al., 2021). Above all, by using both self-report (study 1, affective behavioral response) and electrophysiological measures (study 2, affective neural responses) to collect various indicator of emotion regulation and to evaluate the effectiveness of emotion regulation more objectively, the present study explore whether individuals with AP could use cognitive appraisal strategy to down-regulate their negative emotions more effectively relative to individuals with HP.

## Study 1

### Materials and methods

#### Participants

We conducted an *a priori* power analysis using G*Power, 3.1 (Faul et al., 2007) for multiple linear regression. The effect size was set to detect a small effect (*f* = 0.15), with a statistical power of 0.95 and a significance level of 0.01 to estimate the sample size. According to the power analysis, we needed a minimum of 169 participants. Two hundred and fifty-six college students were recruited through an online portal for undergraduates seeking to participate in the present study. After elimination of 21 randomly responding participants, 235 participants remained in the final sample (186 female, 49 male). The mean age of those in the sample was 21.68 years (*SD* = 3.34). The present study was approved by the local research ethics committee (HR 282-2019). All participants signed an informed consent form.

#### Measures

##### Authentic and hubristic pride-proneness scale

Trait pride was assessed with the 14-item AHPPS (Tracy and Robins, 2007b), including 7 items to assess trait AP and 7 items to assess trait HP. Participants were asked to rate these items on a 7-point Likert-type scale ranging from 1 (not at all) to 7 (very much). Consistent with previous studies (Carver et al., 2010; Cheng et al., 2010; Damian and Robins, 2013), the AHPPS in this study demonstrated high internal reliability for AP (α = 0.87) and HP (α = 0.90). Participants were required to indicate to what extent the currently (or generally) feel in certain ways for the measurement of AP and HP. AP was measured by the extent the participants indicated they felt *accomplished/achieving/confident/fulfilled/productive/self-worth/successful*. HP was measured by the extent they felt *smug/arrogant/conceited/stuck-up/egotistical/pompous/snobbish*. We computed a total score such that high scores correspond to greater tendency toward AP or HP.

##### Effectiveness of negative emotion regulation

The use of specific strategies for regulating negative emotion was measured with the Emotion Regulation Profile-Revised (ERP-R; Nelis et al., 2011). The ERP-R was used to measure participants’ most likely response(s) to nine negative vignettes that describe negative situations eliciting negative emotions (i.e., anger, sadness, fear, shame, guilt, and jealousy). Following the methods of Ortner et al. (2018), for each negative vignette, we asked participants to identify which emotion regulation strategies they might use. The strategies included four adaptive strategies: situation modification (e.g., getting help from a friend to prepare for a presentation), attention reorientation (e.g., thinking about a happy memory), positive reappraisal (e.g., looking for the positive in a situation), and emotion expression (e.g., sharing emotions); and four maladaptive strategies: learned helplessness (e.g., doing nothing to improve a situation), rumination (e.g., focusing on negative thoughts), substance abuse (e.g., using alcohol or smoke to escape a situation), and acting out (e.g., yelling when angry). Participants were required to selected as many strategies as they wished for each scenario. And participants also indicated how effective they thought after they chose these emotion regulation strategies at a general level with their responses to the question, “How much better or worse have efforts to change your thoughts or feelings made you feel?” on a 1 (much worse) to 5 (much better) scale.

Similar to previous study (Ortner et al., 2018), each adaptive strategy chosen was coded with +1 point and each maladaptive strategy chosen was credited −1 point. The total score was calculated by summing the scores for each emotion regulation strategy (computed by summing the adaptive and maladaptive points for negative scenarios) and the score of self-rated strategy effectiveness, and this calculation yielded the total score of down-regulation of negative emotions, with higher scores reflecting higher abilities of emotion regulation of negative emotion. The Cronbach’s alpha for the ERP-R in this sample was 0.78, indicating acceptable internal consistency.

##### Emotion regulation questionnaire

The ERQ consists of 10 items to assess two specific strategies of emotion regulation: cognitive reappraisal (6 items) and expressive suppression (4 items), using a 7-point Likert-type scale from 1 (strongly disagree) to 7 (strongly agree) (Gross and John, 2003). An example of a cognitive reappraisal item is “*When I want to feel more positive emotion or less negative emotion, I change the way I think about the situation.*” An example of an expressive suppression item is “*I did not express my emotions to control them.*” Scores for each scale were calculated by taking the sum of the scores for each item in that scale. High scores on each subscale indicate higher levels of the cognitive reappraisal trait or expressive suppression trait, respectively. In the current sample, the values for Cronbach’s α were acceptable for expressive suppression (α = 0.80) and good for cognitive reappraisal (α = 0.84).

#### Statistical analysis

Missing data analysis showed that the percentage of missing data was low (<5%) and, thus, we used a complete case analysis. Means, standard deviations, and correlations were analyzed prior to running the mediation analysis. Pearson correlation coefficient was used to examine the correlations between the study variables. Table 1 shows the results of descriptive statistics and bivariate correlation.

**TABLE 1:** Descriptive statistics and correlations among variables.

Measure	*M*	*SD*	1	2	3	4	5
1 Authentic pride	32.63	6.66	/				
2 Hubristic pride	24.03	7.91	0.38^**^	/			
3 Reappraisal	30.25	4.95	0.43^**^	0.06	/		
4 Suppression	14.72	4.04	–0.11	0.14*	–0.13	/	
5 Down-regulation of negative emotion	6.93	5.60	0.30^**^	0.02	0.29^**^	–0.38^**^	/

*p < 0.05, **p < 0.01.

The SPSS PROCESS macro developed by Hayes was used to conducted serial mediation analysis (Hayes, 2018). The bootstrapping method with 2,000 resamples of the data was used to test the robustness of mediating effects, with a 95% CI did not contain zero indicating a significant effect (Hayes, 2018). First, the preliminary model (using model 4) was established to initially estimate the association between two facets of pride and the ability of down-regulation of negative emotions, which was meditated by “cognitive appraisal” or “expressive suppression.” Then, we examined whether two kinds of emotion regulation strategies mediated the relation between two faces of pride and the ability of down-regulation of negative emotions, AP and HP were two predictors, the score of down-regulation of negative emotions was outcome (using model 4). In addition, we also tested alternative mediation model, whether the mediating effects of two specific regulation strategies (cognitive reappraisal, expressive suppression) on down-regulation of negative emotions through two facets of pride (using model 4). Sex and age were adjusted in the model.

### Results

#### Bivariate correlations

We calculated Bivariate Pearson’s *r* correlations for the all variables included in the study: AP, HP, cognitive reappraisal, expressive suppression, and down-regulation of negative emotions. Descriptive statistics and bivariate correlations are shown in Table 1. As expected, a significant positive correlation was found between AP and cognitive reappraisal (*r* = 0.43, *p* < 0.01). AP was also positively and significantly related to down-regulation of negative emotions (*r* = 0.30, *p* < 0.01). However, HP was positively related to expressive suppression (*r* = 0.14, *p* < 0.05) but was not significantly related to cognitive reappraisal and down-regulate of negative emotions.

#### Mediation analyses

To test whether the effects of two facets of pride (AP vs. HP) on the ability to regulate negative emotions were mediated by two specific emotional regulation strategies, a multiple mediation model was analyzed with the approach of structural equation modeling (SEM) using SPSS AMOS version 20.0 software. Bootstrapping analysis was used to randomly construct 2,000 samples and conduct parameter estimation. The possible mediation models were tested *via* one separate path analysis in SPSS AMOS software and we only reported the significant indirect or mediational effects in the structural model. According to this contemporary approach, if the 95% bootstrapping confidence intervals from bootstrap samples do not include zero, the mediational model is supported and there is no need to conduct other analyses (Preacher and Hayes, 2004; Hayes, 2009).

**FIGURE 1:**
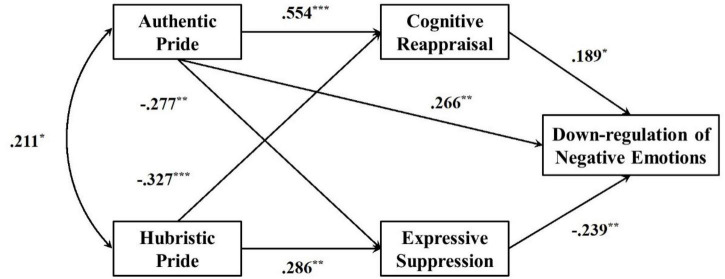
Mediating effect model of two kinds of emotional regulation strategies (cognitive reappraisal; expressive suppression) between two facets of pride and down-regulation of negative emotions. ***p < 0.001, **p < 0.01, *p < 0.05.

Model fit indices included chi-square (χ*^2^*), comparative fit indices (CFI), standardized root mean residual (SRMR), and the root-mean-square error of approximation (RMSEA) and its 90% CI. We used an RMSEA value of ≤0.05, a CFI value ≥ 0.95 as 90% confidence interval (CI) upper limit < 0.095 as indications of good fit (Bentler, 2007). Consisted with Kline (2011), a close approximate fit was indicated by CFI ≥ 0.90 and RMSEA ≤ 0.05. The indirect effects of two facets of pride on the ability to down-regulate negative emotions *via* cognitive reappraisal and expressive suppression were tested. Model fitness measures indicated a good fit (χ*^2^*/df = 1.286, *p* < 0.01, RMR = 0.99, SRMR = 0.051, CFI = 0.958, GFI = 0.873, TLI = 0.951, RMSEA = 0.035, 90% CI = [0.02, 005]). As shown in Figure 1, AP directly predicted down-regulation of negative emotion (β = 0.266, *p* < 0.01, 95% CI = [0.105, 0.425]), significantly and positively predicted cognitive reappraisal (β = 0.554, *p* < 0.001, 95% CI = [0.356, 0.635]), and negatively predicted expressive suppression (β = -0.277, *p* < 0.01, 95% CI = [–0.451, –0.055]). More importantly, cognitive reappraisal partially mediated the effect between AP and down-regulation of negative emotions. The indirect path coefficient was 0.105, 95% CI = [0.002, 0.187]. In addition, expressive suppression partially mediated the effect of AP on down-regulation of negative emotions. The indirect path coefficient was 0.066, 95% CI = [0.011, 0.130].

The results showed that HP had a negative significant direct effect on cognitive reappraisal (β = -0.327, *p* < 0.001, 95% CI = [-0.478, -0.172]), a positive significant direct effect on expressive suppression (β = 0.286, *p* < 0.01, 95% CI = [0.084, 0.468]). HP negatively predicted down-regulation of negative emotions *via* cognitive reappraisal and expressive suppression. The standardized indirect path coefficients were -0.062 and -0.068 with 95% CI = [-0.068, -0.001] and 95% CI = [-0.088, -0.007], respectively.

We also tested the alternative path diagrams. The indirect effects of two specific emotion regulation strategies (cognitive reappraisal vs. expressive suppression) on the ability to down-regulate negative emotions *via* authentic pride and hubristic pride were tested.

**FIGURE 2:**
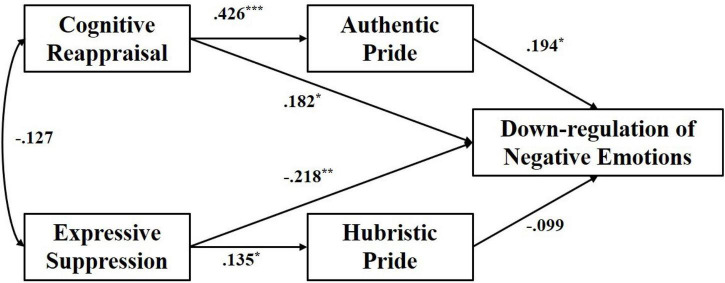
Mediating effect model of two faces of pride (AP; HP) between two kinds of emotional regulation strategies and down-regulation of negative emotions. ***p < 0.001, **p < 0.01, *p < 0.05.

Model fitness measures indicated a good fit (χ*^2^*/df = 1.05, *p* < 0.05, RMR = 0.89, SRMR = 0.048, CFI = 0.931, GFI = 0.845, TLI = 0.921, RMSEA = 0.032). As shown in Figure 2, cognitive reappraisal directly predicted down-regulation of negative emotion (β = 0.182, *p* < 0.05, 95% CI = [0.075, 0.464]), significantly and positively predicted AH (β = 0.426, *p* < 0.001, 95% CI = [0.417, 0.731]), AP had a positively effect on down-regulation of negative emotion (β = 0.194, *p* < 0.05, 95% CI = [0.081, 0. 375]). More importantly, AP partially mediated the effect between cognitive reappraisal and down-regulation of negative emotions. The indirect path coefficient was 0.08, 95% CI = [0.002, 0.146]. In addition, expressive suppression negatively predicted down-regulation of negative emotions (β = −0.218, *p* < 0.01, 95% CI = [−0.598, −0.156]), and positively significant direct effect on HP (β = 0.135, *p* < 0.05, 95% CI = [0.015, 0.416]) (please see Figure 2).

### Discussion

As we expected, we found in study 1 that AP positively predicted negative emotion regulation *via* cognitive reappraisal strategy, whereas HP negatively predicted emotion regulation *via* expressive suppression strategy. Previous studies have shown that AP reflected internal, unstable, controllable attributions (e.g., I did well because I tried hard), whereas HP reflected stable and uncontrollable attributions (e.g., I did well because I am great) (Tracy and Robins, 2007b; Dickens and Robins, 2022). We propose that these differences may help explain these two emotion regulation strategies (cognitive reappraisal vs. expressive suppression) that were associated with individuals with two facets of pride differently. When someone high in AP feels bad or encounters a failure, he/she tends to use cognitive reappraisal strategy (reevaluates a given situation). This strategy allows the person to look for alternatives to cope with the situation in a more adaptive way, which leads to more successful regulation of negative emotions. In contrast, individuals high in HP likely puff themselves up to try to demonstrate their superior natural ability to others, may care about what others think, and have some fear of being negatively judged by others. Thus, individuals with HP may suppress emotional-related behaviors (e.g., making facial expressions) associated with negative emotional responses to avoid being negatively judged by others when encountering negative life events.

These results of study 1 are also in accordance with a number of studies that showed that AP was positively correlated with well-being, but HP was negatively correlated with well-being (Tracy et al., 2009; Orth et al., 2010). Individuals with AP were more likely to employ more effective emotion regulation strategies, leading to more adaptive psychological outcomes, whereas HP positively predicted higher levels of expressive suppression, a maladaptive psychological outcome (Tracy and Robins, 2003, 2014; Tracy et al., 2009; Rogoza et al., 2018). Taken together, these results showed that individuals with trait AP were more likely to habitually use the cognitive reappraisal strategy in everyday life to down-regulate their negative emotions. However, HP was negatively associated with habitual use of cognitive reappraisal and positively associated with habitual use of expressive suppression, resulting in less effective regulation of negative emotions.

In study 1, we examined the relationship between two forms of pride and emotion regulation strategies using a cross-sectional design based on self-report measures. In study 2, therefore, we manipulated the tasks related to emotion regulation with experimental instructions (down-regulate negative emotion vs. up-regulate positive emotion) and further explored whether individuals high in AP could use the cognitive reappraisal strategy more successfully than those high in HP to regulate their emotional reactions. We also recorded the ERPs and measured the LPP as an index of successful emotion regulation processing during cognitive reappraisal. We expected to observe lower emotion intensity ratings and decreased amplitudes of LPP in response to negative pictures in participants with trait AP, but not in participants with HP.

## Study 2

### Materials and methods

#### Participants

Based on previous studies (Qi et al., 2016; Ma et al., 2019), we computed *a priori* power analysis using the G*Power computer program (Faul et al., 2007) to estimate the sample size necessary to achieve an effect size (*f* = 0.20), and a significance level of.0.05 with statistical power (1–β) set at 0.90, using repeated measures analysis of variance (ANOVA) with 1 df. This resulted in an estimated minimum of 66 total participants.

By referring to previous studies on the methods of creating two groups based on self-reported scores of individual difference measurements (Gartland et al., 2021; Wang et al., 2021; Deng et al., 2022; Gong et al., 2022), a total of 70 participants (13 male, 57 female, *M*_*age*_ = 21.42 years, *SD* = 1.16) were selected from 406 college students. The AHPPS was applied during the pre-screening test. Participants with AP scores in the top 25% and HP scores below the 25% percentile were allocated to the AP group, and those with HP scores in the top 25% and AP scores below the 25% percentile were allocated to the HP group. This screening yielded an AP group (37 participants, 6 male, *M*_*age*_ = 22.08 years, *SD* = 1.23) and HP group (33 participants, 7 male, *M*_*age*_ = 20.76 years, *SD* = 1.09). Participants received compensation for their participation. All participants had to fulfill two inclusion criteria: (a) normal hearing and normal/corrected-to-normal vision and (b) no current psychological or psychiatric treatment. Each participant provided an informed consent and the study was approved by the local research ethics committee (HR 282-2019).

#### Affective stimuli materials

Two hundred emotional pictures were selected from the International Affective Picture System (IAPS; Lang et al., 2008). We recruited a new sample of 45 undergraduates (32 female, *M*_*age*_ = 20.46 years, *SD* = 1.48) to rate these pictures on a 9-point scale in terms of valence (1 very unpleasant to 9 very pleasant) and arousal (1 very calm to 9 very excited), including 40 neutral pictures (valence: *M* = 5.21, *SD* = 0.28; arousal: *M* = 3.06, *SD* = 0.29), 80 negative pictures (valence: *M* = 2.19, *SD* = 0.47; arousal: *M* = 6.80, *SD* = 0.75), and 80 positive pictures (valence: *M* = 6.97, *SD* = 0.46; arousal: *M* = 4.87, *SD* = 0.60). For the valence, negative pictures were rated significantly lower than neutral pictures, *t*(118) = -36.80, *p* < 0.001, and positive pictures were rated significantly higher relative to neutral pictures, *t*(118) = 22.26, *p* < 0.001. For the arousal, both positive pictures and negative pictures were rated significantly higher than neutral pictures, *t*(118) = 18.20, *p* < 0.001; *t*(118) = 30.65, *p* < 0.001 and the rating of arousal did not differ between positive pictures and negative pictures, *t*(118) = -1.05, *p* > 05.

#### Emotion regulation task

In the emotion regulation task, participants were instructed to either naturally view emotional pictures (passive view block); reinterpret the cause, outcome, and significance of the pictured events to decrease negative emotions (down-regulation block); or reinterpret the cause, outcome, and significance of the pictured events to increase positive emotions (up-regulation block) with continuous EEG recording. The order of the passive viewing (neutral, negative, positive), negative down-regulation (negative pictures), and positive up-regulation (positive pictures) blocks was fully counterbalanced across participants. For each block there were two 1-min breaks, one halfway through the block and one at the end of the block. The experiment consisted of 20 practice and 160 experimental trials. There were 200 trials in the passive viewing block consisting of 40 neutral, 40 negative, and 40 positive images randomly intermixed. There were 40 trials in the down-regulation block consisting of 40 negative images, and 40 trials in the up-regulation block consisting of 40 positive images.

In the passive viewing block, participants were instructed that they should “just look at the picture carefully and let yourself feel whatever that image makes you feel naturally.” In the down-regulation block, participants were instructed that they try to reinterpret or reevaluate the pictured event to decrease one’s negative emotional response (e.g., imagine that the pictures are just taken from movies or that something good is about to happen). In the up-regulation block, participants were required to attempt to increase their positive emotions by reappraising the meaning of the image (e.g., imagine yourself or a loved one as the central figure in the scene).

**FIGURE 3:**
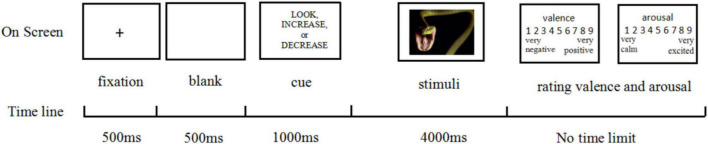
Sample trial sequence of the emotion regulation task.

In each trial, participants were initially presented with a black fixation cross at the center of the screen for 500 ms, which was followed by a 500-ms blank. Then participants saw the cue word “Look,” or the cue words “Increase/Decrease” for 1,000 ms, after which IAPS pictures were then displayed for 4,000 ms; the order of these pictures was fully randomized within each block. After responding to each image according to the trial instructions, participants used the keyboard to rate the level of each image’s pleasantness on a 9-point Likert scale ranging from 1 (very negative) to 9 (very positive) and then rated their emotional arousal on a 9-point Likert scale ranging from 1 (very calm) to 9 (very excited) (see Figure 3). After the regulation task, participants wrote down what they had thought to up and down-regulate their feelings to verify that they had followed the emotion regulation instructions.

#### Electroencephalogram recordings and data reduction

Electroencephalogram data were continuously recorded with a 10-10 system and 64 channels cap, distributed around the head and face, in addition to the scalp electrodes. The analyzer was a GES400 high-density, whole-head recording system combined with Net Station software, both produced by EGI Geodesic. Consistent with previous studies (Li et al., 2008; Harrison and Chassy, 2019). Eye movements were recorded about 1 cm below the left and right eyes. The data were recorded at a sampling rate of 250 Hz and band-pass filtered between 0.1 and 30 Hz. Electrode impedances were kept below 5 KΩ for all electrodes. The recorded signals for all electrodes were referenced to the vertex electrode (Cz). EEG data were re-referenced off-line against the average reference. Stimulus-locked EEG data were segmented offline into 4,200 ms epochs spanning 200 ms pre-stimulus to 4,000 ms post-stimulus.

Independent component analysis (ICA, Delorme et al., 2007) was performed on each participant’s data, and components that were clearly associated with eyeblinks or horizontal eye movements—as assessed by visual inspection of the waveforms and the scalp distributions of the components—were removed. Data exceeding ± 80 μV were rejected and remaining artifacts were manually removed; 3.3% of the trials were excluded from further analyses. Epochs were baseline corrected using the 200-ms pre-stimulus interval. After artifact exclusion, ERP analyses included at least 35 trials per experimental condition. Four participants were excluded because they did not perform the experimental task correctly. In addition, six subjects’ data were excluded because of uncorrectable eye movement artifacts, resulting in the final sample of 31 participants (6 male) in the AP group and 29 participants (5 male) in the HP group.

#### Data analysis

For the ERP data, the mean amplitude of an LPP was an ERP-dependent measure. Based on previous studies (Foti and Hajcak, 2008; Krompinger et al., 2008; Kinney et al., 2019), electrode pooling was created performed (P_*Z*_, P1, P2, P3, P4, P7, P8) to evaluate the activity with the LPP. According to previous studies (Parvaz et al., 2012; Kinney et al., 2019) in which the duration of time that pictures were displayed was more than 1,000 ms, LPP was calculated as the mean amplitude in three time windows: early (500–1,000 ms post-picture onset), middle (1000–2,500 ms post-picture onset), and late (2,500–4,000 ms post-picture onset) to better understand the time course of the emotion regulation process. ERP waveforms locked to the onset of emotional pictures were created for each of the five experimental conditions: negative–viewing, positive–viewing, neutral–viewing, negative–down-regulation, and positive–up-regulation.

### Results

#### Authentic and hubristic pride-proneness scale test scores

To investigate grouping effectiveness, we further analyzed the participants’ responses under two groups based on the self-reported scores of HP sub-scale and those of AP sub-scale respectively. These results revealed that the mean AP scores of AP group (5.96 ± 1.19) were higher than that of HP group (4.05 ± 1.26) significantly, *t*(68) = 6.54, *p* < 0.001, Cohen’s *d* = 1.56. And the mean HP scores of HP group (4.72 ± 1.68) were higher than that of AP group (2.86 ± 1.56), *t*(68) = 4.83, *p* < 0.001, Cohen’s *d* = 1.15. These results verified that the grouping was effective.

#### Self-reported results

We analyzed the mean scores for the subjective rating of emotional valence and arousal of the AP and HP groups in different emotion regulation conditions (down-regulation and up-regulation) with repeated measures ANOVA.

For the condition of down-regulation of negative emotions, mean scores for the subjective rating of emotional valence and arousal were analyzed by two (Group: AP vs. HP) × 2 (Condition: “passive viewing negative” vs. “down-regulation negative”) repeated measures ANOVAs: one for valence and one for arousal. Analysis of the valence revealed a main effect of condition on valence ratings, *F*_(1,68)_ = 298.77, *p* < 0.001, η_*p*_^2^ = 0.83, indicating that the valence ratings of negative pictures were significantly lower in the down-regulation condition (*M* = 3.96, *SD* = 0.55) relative to the passive viewing condition (*M* = 2.29., *SD* = 0.58). The main effect of group was not significant, *F*_(1,68)_ = 1.11, *p* = 0.30, the two-way interaction between group × condition was significant, *F*_(1,68)_ = 5.03, *p* < 0.05, indicating that the valence ratings of negative pictures for AP group (*M* = 4.13, *SD* = 0.44) were higher than HP group (*M* = 3.80, *SD* = 0.59) in down-regulation condition, *t*(68) = 2.46, *p* < 0.05, Cohen’s *d* = 0.63, however, there was no significant difference on the scores for the self-rating of valence between AP group (*M* = 2.23, *SD* = 0.57) and HP group (*M* = 2.35, *SD* = 0.59).

**FIGURE 4:**
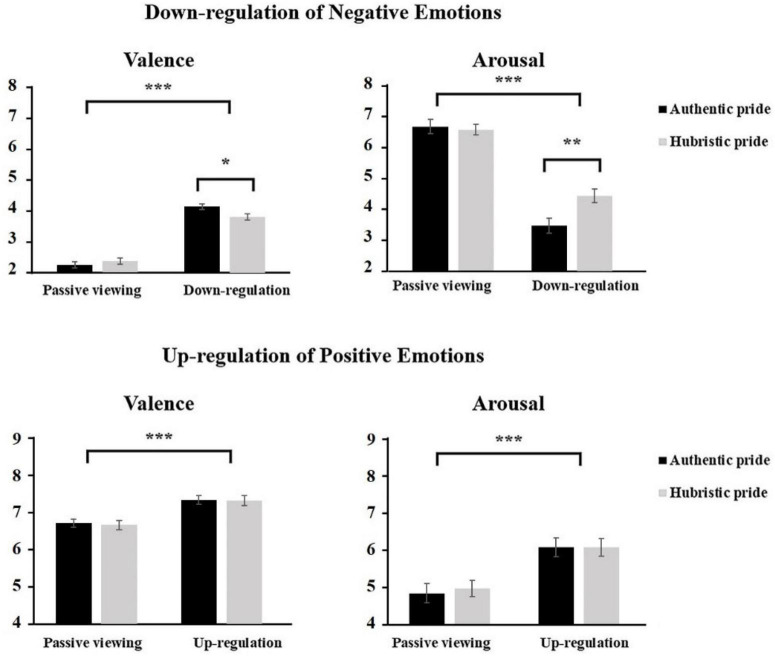
The mean scores of valence rating and arousal rating of different emotional regulation conditions in AP group and HP group. ***p < 0.001, **p < 0.01, *p < 0.05.

For the arousal ratings of negative pictures, the main effect of group was not significant, *F*_(1,68)_ = 3.27, *p* = 0.08, η_*p*_^2^ = 0.05. However, the main effect of condition was significant, *F*_(1,68)_ = 197.72, *p* < 0.001, η_*p*_^2^ = 0.77, indicating that the arousal ratings of negative pictures was significantly lower in the down-regulation condition (*M* = 3.96, *SD* = 1.35) relative to the passive viewing condition (*M* = 6.58, *SD* = 1.10). Critically, the two-way interaction of group × condition was also significant, *F*_(1,68)_ = 8.10, *p* < 0.01, η_*p*_^2^ = 0.12. Simple effect analysis showed that the arousal ratings reported by the AP group (*M* = 3.46, *SD* = 1.30) were significantly lower than those reported by the HP group (*M* = 4.42, *SD* = 1.25) in the down-regulation condition, *t*(68) = −2.98, *p* < 0.01, Cohen’s *d* = 0.75. However, the difference between the AP group (*M* = 6.63, *SD* = 1.24) and HP group (*M* = 6.53, *SD* = 0.97) was not significant in the passive negative viewing condition, *t*(68) = 0.36, *p* = 0.72 (see Figure 4). These results suggest that relative to individuals in the HP group, individuals in the AP group could more effectively use the cognitive reappraisal strategy to down-regulate negative emotional arousal.

For the condition of up-regulation of positive emotions, we also conducted two (Group: AP vs. HP) × 2 (Condition: “passive viewing positive” vs. “up-regulation positive”) repeated measures ANOVAs on mean scores for the subjective ratings of emotional valence and arousal: one for valence and one for arousal. For the valence self-ratings of positive pictures, there was a significant main effect of condition (up-regulation positive/passive positive viewing), *F*_(1,68)_ = 94.59, *p* < 0.001, η_*p*_^2^ = 0.61, indicating that the valence of positive pictures in the up-regulation condition (*M* = 7.31, *SD* = 0.72) was significantly higher relative to that of the passive-viewing condition (*M* = 6.67, *SD* = 0.64). However, neither the main effect of group nor the two-way interaction of group × condition was significant, *Fs* < 1. For the arousal of positive pictures, the main effect of condition was significant, *F*_(1,68)_ = 86.53, *p* < 0.001, η_*p*_^2^ = 0.59, indicating that the arousal associated with positive images in the up-regulation condition (*M* = 6.06, *SD* = 1.34) was significantly higher than that associated with the passive-viewing condition (*M* = 4.89, *SD* = 1.33). Neither the main effect of group, *F*_(1,68)_ = 0.05, *p* = 0.82 nor the interaction of group × condition, *F*_(1,68)_ = 0.25, *p* = 0.62, was significant (see Figure 4).

#### Event-related potential results

**FIGURE 5:**
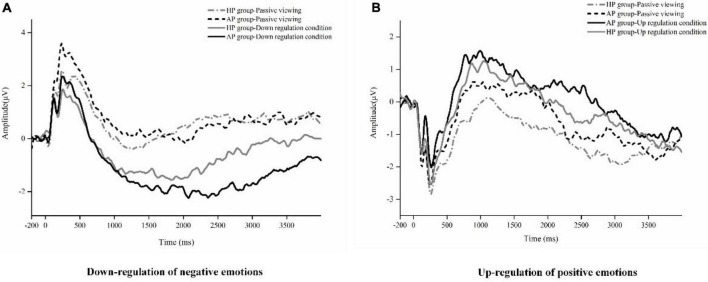
Mean waveforms by condition in the AP and HP groups. **(A)** Average stimulus-locked ERPs for two experimental conditions (down-regulation, passive viewing) in the AP and HP groups. **(B)** Average stimulus-locked ERPs for two experimental conditions (up-regulation, passive viewing) in the AP and HP groups.

**FIGURE 6:**
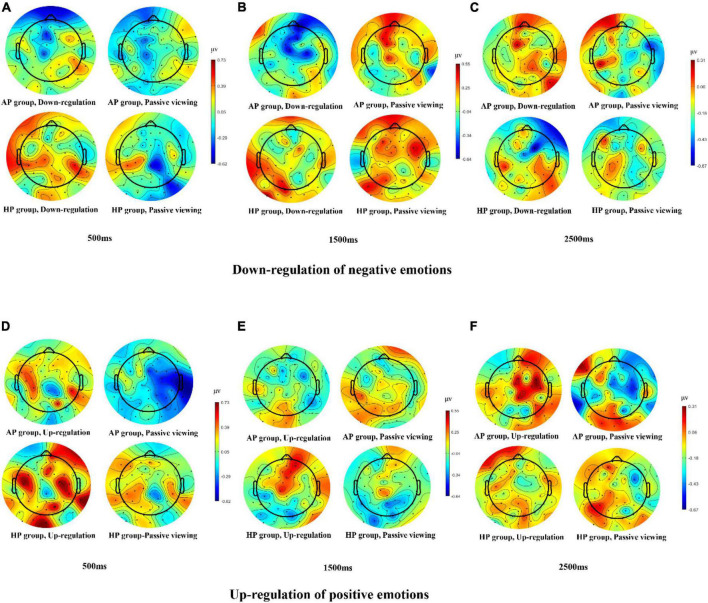
The topographic maps of LPP amplitudes for different conditions. **(A–C)** Topographic maps of LPP amplitudes for down-regulation and passive viewing conditions in AP and HP groups at 500 ms, 1500 ms, and 2500 ms, respectively. **(D–F)** Topographic maps of LPP amplitudes for up-regulation and passive viewing conditions in AP and HP groups at 500 ms, 1500 ms, and 2500 ms, respectively.

For the down-regulation of negative emotions, 2 (Group: AP vs. HP) × 2 (Condition: passive viewing negative pictures vs. down-regulation of negative pictures) × 3 (Time window: early [500–1,000 ms], middle [1,000–2,500 ms], late [2,500–4,000 ms]) mixed ANOVA tests were conducted for LPP amplitudes. There was a significant two-way interaction between group and condition, *F*_(1,58)_ = 4.60, *p* < 0.05, η_*p*_^2^ = 0.10, indicating that the amplitude of LPP was significantly lower in the AP group (*M* = -3.38, *SD* = 3.26) compared to the HP group (*M* = -1.53, *SD* = 3.40) during down-regulation of negative emotions (*p* < 0.05), but not in the passive viewing negative pictures condition (AP group, *M* = -0.16, *SD* = 2.94; HP group, *M* = -0.75, *SD* = 3.75). Additionally, we found a significant main effect of condition, *F*_(1,58)_ = 12.27, *p* < 0.01, η_*p*_^2^ = 0.22, suggesting that the amplitude of LPP in the down-regulation of negative pictures condition was significantly lower relative to that of passive viewing negative stimuli condition. The main effect of time window was also significant, *F*_(2,57)_ = 17.26, *p* < 0.001, indicating that the amplitude of LPP was larger in the early stage than in the middle stage (*M* = -2.42, *SD* = 3.52) and the late stage (*M* = -1.30, *SD* = 3.50). No other significant effect was found, *Fs* < 1 (see Figures 5, 6).

For the up-regulation of positive emotions, we also conducted a 2 (Group: AP vs. HP) × 2 (Condition: passive viewing positive stimuli vs. up-regulation) × 3 (Time window: early, middle, late) mixed ANOVA tests on LPP amplitudes. The main effect of condition was significant, *F*_(1,58)_ = 4.60, *p* < 0.05, indicating that the amplitude of LPP for the up-regulation condition (*M* = 0.24, *SD* = 3.00) was significantly larger than that of the passive viewing condition (*M* = -0.95, *SD* = 2.87). No other significant main or interaction effects was found for LPP amplitudes, all *Fs* < 2 (see Figures 5, 6).

### Discussion

Consistent with our hypothesis, the results of study 2 revealed that, in contrast to individuals with HP, individuals with AP rated negative pictures as less threatening during down-regulation of negative stimuli, indicating that individuals with AP utilized the cognitive reappraisal strategy to decrease negative emotion responses more successfully than did those with HP. We also observed lower LPP amplitudes associated with negative pictures during cognitive reappraisal in the AP group than in HP group. The LPP is a classic and sensitive ERP index for successful negative emotion regulation during reappraisal (Dennis and Hajcak, 2009; Hajcak et al., 2010). The increased LPP amplitude reflects enhanced emotion processing, and the reduced LPP amplitude represents a corresponding decline in emotional experience and related emotion processing (Weinberg and Hajcak, 2010; Harrison and Chassy, 2019; Pan et al., 2019). Therefore, the use of experimental emotional regulation tasks in study 2 further revealed that individuals with AP utilized cognitive reappraisal strategy to regulate their negative emotions more effectively relative to individuals with HP which was also found in study 1.

According to the theory of the two facets of pride (Tracy and Robins, 2007b,^2014^), AP is positively related to self-esteem, whereas HP may be a basis for narcissism. AP is associated with unstable, controllable attributions to success. Individuals with AP hold the opinion that every coin has two sides, and, therefore, tend to reinterpret the content of negative stimuli to decrease their negative emotional reactions. In contrast, individuals with HP may display their exaggerated pride to others to protect a fragile ego or their low self-esteem, resulting in actively suppressing their negative feelings to negative stimuli (Tracy and Robins, 2003, 2007b,^2014^). This could be thought as a type of defensive response to protect their self-esteem. Additional evidence indicated that HP was related to decrements in voluntary attentional control or reduction in delayed gratification (Carver et al., 2010; Ho et al., 2016), which may be reasons for unsuccessful reinterpretation of the meaning of an emotional stimulus.

For the up-regulation of positive emotion condition, significant differences in neither self-report measures nor the ERP results were detected. We inferred that individuals with AP and HP both preferred reward or sought to experience pleasure (Kong et al., 2018); therefore, individuals with AP and HP could accomplish up-regulation of positive emotions to the same extent.

## General discussion

Emotion regulation is essential for the state of an individual’s mental health (e.g., Henry et al., 2016) and well-being (e.g., Haga et al., 2009). However, most previous research focused on the precise nature of the emotion dysregulation in emotion disorders (e.g., Werner et al., 2011; Kinney et al., 2019; Pan et al., 2019). There has been limited research into the differential role of two forms of pride on emotion regulation strategies in healthy adults. In two studies, we investigated the differential effects of two forms of pride on the effectiveness of negative emotion regulation. Using self-reported measures, the results of study 1 showed high AP was associated with the habitual use of the cognitive reappraisal strategy more frequently in daily life. In addition, self-reported use of cognitive reappraisal mediated the link between AP and down-regulation of negative emotions. Structural equation models showed that HP was more likely to be positively associated with habitual use of the expressive suppression strategy in everyday life, leading to unsuccessful down-regulation of negative emotions. In study 2, we manipulated emotion regulation tasks with experimentally instructed conditions and found that individuals with AP used the instructed cognitive reappraisal strategy to decrease negative emotions more effectively compared to individuals with HP, which was not only reflected in lower self-reported emotional arousal but also in lower amplitudes of the LPP. Our results indicate that a healthy individual’s initial tendency to experience distinct trait pride (authentic vs. hubristic) could also be differentially associated with the cognitive reappraisal strategy.

According to the authentic/hubristic model of pride (Tracy and Robins, 2007b), two distinct facets of pride are characterized by distinct ways of appraising the causes of achievement. Individuals with AP with stable and genuine self-esteem attribute success to controllable/unstable reasons (e.g., one’s hard work). In contrast, those with HP attribute success to uncontrollable/stable reasons (e.g., one’s superior natural ability); as a result, individuals with elevated HP might show high sensitivity to social evaluations of themselves. We thought this difference might explain the findings of the current study. AP tends to be positively related to the cognitive appraisal strategy, which reflects a controllable/unstable explanation for emotional events, whereas the stable attribution style associated with HP is also associated with the habitual use of the expressive suppression strategy. In addition, previous studies have proved that cognitive reappraisal was closely related to cognitive control (Moser et al., 2010; McRae et al., 2012; McRae and Gross, 2020). Furthermore, evidence shows that the two processes of cognitive reappraisal and cognitive control both recruited the activation of the prefrontal cortex (McRae and Ochsner, 2008; Drabant et al., 2009). Therefore, successful cognitive reappraisal may involve the effective process of cognitive control. A few studies have demonstrated that AP promoted the process of cognitive control, whereas HP undermined it and might have been related to impulsiveness (Carver et al., 2010; Ho et al., 2016; Van Doren et al., 2019). Based on these studies, it seems that two facets of pride can be differently related to cognitive reappraisal, and the present research is among the first to test whether two specific positive self-oriented emotions can affect negative emotion regulation differentially. And the finding of study 1 also found that cognitive reappraisal significantly and positively predicted AH, and expressive suppression significantly and positively predicted HP in the alternative path. Considering that study 1 was a cross-sectional design, it remains unknown whether AP could result in utilizing cognitive reappraisal strategy more frequently.

The results of study 2 further demonstrated that during down-regulation of negative emotions, the amplitude of LPP elicited by negative images was reduced in the AP group relative to that in the HP group. The observed decrease in LPP amplitude is consistent with previous studies that have shown that the LPP is sensitive to regulation of negative emotions *via* (instructed) cognitive reappraisal (Hajcak et al., 2010, 2012; Olatunji et al., 2017; Harrison and Chassy, 2019; Bartolomeo et al., 2020; Norris and Wu, 2021). Attenuation of the LPP amplitude during down-regulation of negative emotions reflects the decreased emotional intensity as a result of cognitive reappraisal, which represents successful regulation of negative emotions (Foti and Hajcak, 2008; Dennis and Hajcak, 2009; Kinney et al., 2019). In sum, the present study provided preliminary evidence that individuals with AP regulated negative emotions more successfully than did those with HP and the decrease of LPP amplitude might reflect the electro-cortical mechanism underlying this mental process.

Our results are consistent with a growing body of literature implicating AP in more adaptive outcomes, including facilitated delayed gratification, more effective leadership behaviors, higher achievement, genuine self-esteem, helping behavior, high level of mental health and well-being, and lower depressive symptoms (Tracy and Robins, 2007b; 2009; Beard et al., 2017; Van Doren et al., 2019; Yeung and Shen, 2019), whereas HP is implicating in maladaptive outcomes, including aggression, anxiety, and depression (Orth et al., 2010; Weidman et al., 2015; Brosi et al., 2016; Cohen and Huppert, 2018). Our findings are also consistent with a recent meta-analysis that suggested that AP and HP are empirically distinct constructs that often align in opposite ways with personality and related variables, with AP exhibiting associations that suggest better psychological health than those exhibited by HP (Dickens and Robins, 2022). Our findings also provide a potential explanation for the results reported in previous studies. When individuals with HP feel negative emotions, they cannot reinterpret the meaning of a negative stimulus with a positive opinion. On the contrary, they have a habitual tendency to use the expressive suppression strategy associated with negative social and emotional consequences, resulting in poorer mental health, impulsive/aggressive behavior, or adverse emotional states (Dryman and Heimberg, 2018; Bedwell et al., 2019; Chen et al., 2020).

During the experimental task of up-regulation of positive emotions using the cognitive reappraisal strategy in study 2, neither the self-reported results nor the ERP results were significantly different between the AP and HP groups. There are several reasons that account for this. Firstly, individuals tend to use the cognitive reappraisal strategy to down-regulate negative emotional reactions instead of up-regulation of positive emotions in daily life (Harrison and Chassy, 2019; Troy et al., 2019; Kneeland et al., 2020; Yeung and Wong, 2020). Secondly, considering that these two forms of pride are both positive self-conscious positive emotions, individuals with AP and HP might value reward and pursue positive emotional experiences to the same extent; therefore, it is possible that individuals with these two traits of pride have the same potential to increase positive emotions effectively (Kong et al., 2018). Thirdly, although previous studies indicated that up-regulation of positive emotions was indeed related to subjective well-being (Shiota, 2006; Quoidbach et al., 2015), the ability to down-regulate negative emotions might play a more important role in successful emotion regulation or maintaining optimal mental states (Ortner et al., 2018).

Several limitations of the current study should be acknowledged. Firstly, the present study revealed the time course of emotion regulation in individuals with different trait pride types; however, EEG has relatively poor spatial resolution. Future fMRI studies using these paradigms could reveal the specific neural circuits contributing to the LPP difference between those with AP and HP observed in this study. Secondly, we explored the differential relations of these two forms of trait pride to emotion regulation strategies in healthy undergraduates. Future research should further explore the associations between emotion regulation and AP and HP among individuals with mental disorders. Some studies have shown that children with autism spectrum disorders with more severe symptoms are more prone to having HP (Davidson et al., 2017, 2018). Depressive and anxious symptoms were also related to lower AP (Tang-Smith et al., 2015). Thirdly, according to previous studies (Cao et al., 2020; Xiao et al., 2021), it might helpful to exclude individuals with failed reappraisal to further explore the relationship between two facets of pride and emotion regulation strategies. Fourthly, in study 2, we did not measure personality straits variables (such as self-esteem, narcissism and self-control), observed patterns might to some extent be caused by these individual differences. Therefore, future studies should control these variables. Fifthly, it is also important to note that the gender ratio of the participants was unbalanced in the present study. Thus, future research in this area needs more representative samples to increase the validity of our results. Finally, the use of a cross-sectional design in study 1 does not allow to make causal inferences precludes causal inference. The cross-sectional design is well-suited for testing assumptions about the relationships of two facets of pride and emotion regulation strategies. However, such design could not separate between a presumed cause and its possible effect, it remains unclear whether individuals with AP could lead to greater use of cognitive reappraisal to down-regulate negative emotions. And, an additional limitation is the potential for social desirability bias in study 1, the two facets of pride may involve different levels of social desirability biases using self-report method, with more social desirability of the AP items relative to the HP items (e.g., “successful” vs. “arrogant”), which can cause that participants might tend to report he/she is an individual with AP trait.

## Conclusion

The current study is unique in that it explores whether two facets of pride (authentic vs. hubristic) are differentially related to the cognitive reappraisal strategy. Across two studies, we found converging evidence that individuals with AP utilized cognitive reappraisal strategy (under experimentally instructional conditions; spontaneously) more successfully to down-regulate negative emotions than those with HP, whereas HP is more likely to be associated with the spontaneous expressive suppression strategy. These findings contribute to the theoretical value of examining specific positive emotions. The present research provides an explanation for why two forms of trait pride were associated with different mental health states from the perspective of emotion regulation.

## Data availability statement

The raw data supporting the conclusions of this article will be made available by the authors, without undue reservation.

## Ethics statement

The studies involving human participants were reviewed and approved by the Ethics Committee of East China Normal University. The patients/participants provided their written informed consent to participate in this study.

## Author contributions

YW and DL: concept and design of study. DL, JB, and YW: data acquisition, analysis, and interpretation. YW, DL, XZ, and FZ: drafting the work or revising it critically for important intellectual content. YW: agreement to be accountable for all aspects of the work in ensuring that questions related to the accuracy or integrity of any part of the work were appropriately investigated and resolved. All authors: final approval of the version to be published.
